# 

**Published:** 1848-07

**Authors:** 


### CONTENTS.

### LIBRARY.

PAGE.

The Youth's Dentist, or the Way to have Sound and Beautiful Teeth. By J. R. Duval, Dentist, . . . . . . . . 1

### JOURNAL.

Article I.—A Course of Lectures on Dental Physiology and Surgery. By John Tomes, Esq., . . . . . . . 313

Article II.—Case of Spontaneous Destruction of the Alveoli, Second Bicuspis and First Molaris. Reported by S. M. Sheppard, D. D. S., . . . . . . . . . . . 350

Article III.—Reports in Brief of Four Cases in Surgery, and One in Obstetrics in which Chloroform was Beneficially Used. By M. Z. Kreider, M. D., . . . . . . . . . . 352

Article IV.—Neuralgia.—Case of Miss E. R. L. By J. Lee, M. D. 354

Article V.—On the Management of Irregular Dentition. By J. B. Mitchell, M. D., . . . . . . . . . . 335

Article VI.—Operations on the Jaws, with the Results of Thirteen Cases. By Paul F. Eve, M. D., . . . . . . . . 367

Article VII.—A Thesis on Tic Douloureux, or Neuralgia Faciei. Presented by John McCalla, D. D. S., . . . . . . . 374

Article VIII.—Fracture of the Nasal Bones, . . . . . . 383

Article IX.—Attraction of Cohesion in Gold Fillings. By J. W. Crane, M. D., . . . . . . . . . . . 392

Article X.—Letter from G. F. J. Colburn, . . . . . . 394

### MISCELLANEOUS NOTICES.

American Society of Dental Surgeons, . . . . . . . 395

American Medical Association, . . . . . . . . . 396

